# Supplementary material for: Cloud BioLinux: pre-configured and on-demand bioinformatics computing for the genomics community
Source: BMC Bioinformatics. 2012 Mar 19;13:42. doi: 10.1186/1471-2105-13-42 (PMC3372431; doi:10.1186/1471-2105-13-42)
Supplement: Additional file 1 — Supplementary 1 Cloud BioLinux software documentation in the form of a mini, self-contained website. Users need to download and uncompress the .zip file, and open through a web browser the "index.html" file available on the main directory. (ZIP 1823 kb). [file 1471-2105-13-42-S1.ZIP › Cloud-BioLinux-Package-Documentation/docs/bioperl.html]

Bio-Linux Software Documentation Pages

Back to search form

## bioperl

|  |  |
| --- | --- |
| Name | bioperl |
| Description | **bioperl** is effectively Perl modules for bioinformatics  **bioperl** was officially organized in 1995 and existed informally for several years prior. The Bioperl Project is an international association of developers of open source Perl tools for bioinformatics, genomics and life science research.  Facilitated by the Open Bioinformatics Foundation, the bioperl developers work closely with friends and colleagues across many projects including biojava.org, biopython.org, DAS, bioruby.org, biocorba.org, EnsEMBL and EMBOSS.  The Bioperl server provides an online resource for modules, scripts, and web links for developers of Perl-based software for life science research. The bioperl project can also provide web, FTP and CVS space for individuals and organizations wishing to distribute or otherwise make freely available standalone scripts & code. |
| Homepage | http://bio.perl.org/ |
| Remote Documentation | http://bio.perl.org/Core/Latest/modules.html |

|  |
| --- |
| Please see Remote Documentation |
